# Intranasal insulin in Alzheimer disease (diabetes *in situ*?): a systematic review and meta-analysis

**DOI:** 10.1590/1980-5764-DN-2024-0191

**Published:** 2025-04-07

**Authors:** Luís Jesuíno de Oliveira Andrade, Gabriela Matos, Luís Matos de Oliveira

**Affiliations:** 1 Universidade Estadual de Santa Cruz, Departamento de Saúde, Ilhéus BA, Brazil.; 2 Centro Universitário UniFTC, Faculdade de Medicina, Salvador BA, Brazil.; 3 Escola Bahiana de Medicina e Saúde Pública, Salvador BA, Brazil.

**Keywords:** Alzheimer Disease, Insulin, Administration Intranasal, Systematic Review, Meta-Analysis, Doença de Alzheimer, Insulina, Administração Intranasal, Revisão Sistemática, Metanálise

## Abstract

Alzheimer disease (AD) is a neurodegenerative disorder. Evidence suggests that AD shares pathophysiological similarities with type 2 diabetes. Intranasal insulin (INI) has emerged as a potential therapeutic approach for AD by directly targeting the brain and modulating insulin signaling pathways.

**Objective:**

To evaluate the efficacy and safety of INI therapy for AD through a systematic review and meta-analysis of randomized controlled trials.

**Methods:**

A search of electronic databases, including PubMed, Web of Science, Scopus, and Embase, was conducted to identify relevant studies published up to June 2024. Inclusion criteria encompassed peer-reviewed original research articles focused on humans, investigating the therapeutic effects of INI administration on cognitive impairment associated with AD, and reporting quantitative data on cognitive outcomes, biomarkers, or pathological markers relevant to AD. A meta-analysis was conducted to quantitatively synthesize the effects of INI on cognitive outcomes.

**Results:**

A total of 647 articles were identified, and eight studies met the inclusion criteria. The overall odds ratio was 3.75 (95%CI 1.49–9.40). The test for overall effect showed a statistically significant difference (p<0.05). However, the I^2^ value indicated a high level of heterogeneity (85.5%), suggesting significant variability among the studies.

**Conclusion:**

While the current data is not yet conclusive enough to definitively establish INI as a standard treatment for AD, the evidence supporting its safety, efficacy, and reduced risk of systemic side effects suggests potential cognitive benefits for improving global cognition in patients with AD.

## RESUMO.

A Doença de Alzheimer (DA) é um transtorno neurodegenerativo. Evidências sugerem que a DA compartilha semelhanças fisiopatológicas com o diabetes tipo 2. A insulina intranasal (IIN) surgiu como uma abordagem terapêutica potencial para a DA, agindo diretamente no cérebro e modulando as vias de sinalização da insulina.

**Objetivo:**

Avaliar a eficácia e a segurança da terapia com IIN para a DA por meio de uma revisão sistemática e metanálise de ensaios clínicos randomizados.

**Métodos:**

Realizou-se uma busca nas bases de dados eletrônicas PubMed, Web of Science, Scopus e Embase, para identificar estudos relevantes publicados até junho de 2024. Critérios de inclusão: artigos originais de periódicos revisados por pares, focados em humanos, que investigassem os efeitos terapêuticos da administração de IIN no comprometimento cognitivo associado à DA e que relatassem dados quantitativos sobre resultados cognitivos, biomarcadores ou marcadores patológicos relevantes para a DA. Uma metanálise foi realizada para sintetizar quantitativamente os efeitos da IIN nos desfechos cognitivos.

**Resultados:**

Foram identificados 647 artigos e oito estudos atenderam aos critérios de inclusão. A razão de chances geral foi de 3,75 (IC95% 1,49–9,40). O teste para o efeito global mostrou diferença estatisticamente significativa (p<0,05). O valor de I^2^ indicou alta heterogeneidade (85,5%), sugerindo variabilidade significativa entre os estudos incluídos.

**Conclusão:**

Embora os dados atuais ainda não sejam conclusivos o suficiente para estabelecer definitivamente a IIN como tratamento padrão para a DA, as evidências que apoiam sua segurança, eficácia e redução do risco de efeitos colaterais sistêmicos sugerem um potencial benefício para melhorar a cognição global em pacientes com DA.

## INTRODUCTION

Alzheimer disease (AD) is a progressive neurodegenerative disorder characterized by the loss of memory, language, and other cognitive functions. It is the leading cause of dementia, accounting for 60-80% of cases^1^. The pathophysiology of AD is complex and not fully understood. However, two key neuropathological hallmarks are consistently observed: amyloid plaques and neurofibrillary tangles. Amyloid plaques consist of amyloid-beta (Aβ) protein, while neurofibrillary tangles are composed of tau protein. These abnormal protein aggregates are believed to disrupt normal brain function and result in neuronal death^2^. There is currently no cure for AD, and existing treatments are only able to manage symptoms temporarily. However, significant research efforts are underway to develop new treatments and therapies. Promising approaches include targeting Aβ and tau aggregation, modulating neuroinflammation, and enhancing neurogenesis^3^. While challenges remain, there is hope that AD may one day become a preventable and treatable condition.

Brain insulin resistance (BIR) refers to the impaired ability of brain cells to respond to insulin. While insulin is primarily known for its role in peripheral glucose homeostasis, it also plays a critical role in brain functions such as memory, cognition, and learning^4^. BIR has been implicated in the pathogenesis of several neurodegenerative diseases, including AD^5^. In AD, BIR is associated with increased amyloid plaque deposition and tau neurofibrillary tangles, two hallmark neuropathological features of the disease^6^. The mechanisms underlying BIR in the brain are complex and not fully understood. However, factors such as chronic hyperglycemia, obesity, and inflammation are believed to contribute^7^. Current therapeutic approaches for BIR in the brain remain limited.

Intranasal insulin (INI) administration has been proposed as a potential therapeutic strategy for AD, based on the hypothesis that brain insulin resistance contributes to its pathogenesis. INI for AD has emerged as a promising therapeutic approach due to its ability to directly target the brain and modulate insulin signaling pathways. Bypassing the blood-brain barrier allows INI to deliver insulin directly to brain cells, maximizing its therapeutic effect while minimizing systemic side effects^8^. The mechanisms underlying the beneficial effects of INI in AD are multifaceted. INI promotes glucose uptake and energy metabolism in neurons, modulates neuroinflammation, and stimulates neurogenesis, potentially counteracting neuronal loss in AD^9^.

Increasing evidence suggests that AD shares pathophysiological similarities with type 2 diabetes, leading to the conceptualization of AD as "diabetes *in situ*" within the brain. By crossing the blood-brain barrier, INI therapy allows for the delivery of insulin directly to brain cells, maximizing its effect on the brain and with minimal systemic side effects. The aim of this study was to evaluate the efficacy of INI therapy for AD through a systematic review and meta-analysis of randomized clinical trials (RCTs).

## METHODS

### Study design, protocol, and registration

To ensure transparency and methodological rigor, the protocol for this systematic review was prospectively registered on the International Prospective Register of Systematic Reviews (PROSPERO): CRD42024560578. This public record, accessible at https://www.crd.york.ac.uk/prospero/#, outlines the planned search strategy, inclusion/exclusion criteria, and data extraction methods employed in the review. Furthermore, the review adhered to the established Preferred Reporting Items for Systematic Reviews and Meta-Analyses (PRISMA) guidelines throughout the review process^10^, and included RCTs that comprehensively investigated the literature on the potential efficacy of INI for AD.

### Literature search strategy

A comprehensive search was conducted across multiple electronic databases, including PubMed, Web of Science, Scopus, and Embase, to identify relevant studies published up to June 2024. The search strategy employed a combination of controlled vocabulary and free-text terms related to AD and INI therapy. Boolean operators (AND, OR, NOT) were utilized to refine the search and maximize the retrieval of relevant studies. Specific search terms and variations included: ("alzheimer disease"[MeSH Terms] OR ("alzheimer"[All Fields] AND "disease"[All Fields]) OR "alzheimer disease"[All Fields]) AND ("intranasal"[All Fields] AND ("insulin"[MeSH Terms] OR "insulin"[All Fields])). To ensure thoroughness, references of included studies and relevant review articles were also reviewed.

### Inclusion and exclusion criteria

Studies were included if they met the following criteria: Original research articles published in peer-reviewed journals; Focused on humans; Investigated the therapeutic effects of INI administration on cognitive impairment associated with AD or diabetes; Reported quantitative data on cognitive outcomes, biomarkers, or pathological markers relevant to AD or diabetes.

Studies were excluded if they were review articles, editorials, commentaries, case reports, or abstracts lacking sufficient data. Studies focused solely on peripheral metabolic effects without evaluating cognitive or neurological outcomes were also excluded.

### Data extraction and synthesis: study quality assessment

To ensure methodological rigor and transparency, the Cochrane risk-of-bias tool version 2 (ROB2) was employed to evaluate the included RCTs^11^. This standardized tool facilitates a systematic assessment of potential bias across critical domains that could influence study outcomes. These domains encompass: the generation of random sequences to prevent allocation predictability; the implementation of allocation concealment to mask treatment assignment until intervention initiation; the blinding of participants, personnel administering interventions, and outcome assessors to minimize performance and detection bias; the extent of missing outcome data to evaluate the potential for attrition bias; the selective reporting of pre-specified outcomes to safeguard against reporting bias; and the exploration of any other potential sources of bias not captured within the aforementioned domains. Through this comprehensive evaluation using ROB2, the risk of bias impacting the authors’ conclusions was categorized as either "low risk," "some concerns," or "high risk."

Relevant data were extracted using a standardized data extraction form, including study characteristics (authors, publication year, study design, and sample size), participant demographics, details of INI administration (dose, frequency, duration), cognitive assessment tools (The Alzheimer's Disease Assessment Scale-Cognitive Subscale (ADAS-Cog) score and Activities of Daily Living Scale (ADCS-ADL)), and main outcomes.

For studies with available data, a meta-analysis was conducted to quantitatively synthesize the effects of INI on cognitive outcomes. Standardized mean differences and 95% confidence interval (CI) were calculated for continuous outcomes, while odds ratio (OR) and 95% CI were calculated for dichotomous outcomes. Random-effects models were employed to account for potential heterogeneity between studies. Subgroup analyses were conducted based on the type of population (AD or diabetes) and the specific cognitive domain assessed.

### Statistical analysis

The meta-analysis was conducted using METAANALYSISONLINE (https://metaanalysisonline.com/)^12^, an online statistical tool that facilitated the generation of forest plots and funnel plots. Heterogeneity among studies was assessed using the Q statistic and the I^2^ index, with I^2^ values of 25, 50, and 75% were interpreted as indicating low, moderate, and high heterogeneity, respectively. Publication bias was evaluated through visual inspection of funnel plots and Egger's regression test. Sensitivity analyses were conducted to assess the robustness of the results, excluding studies with a high risk of bias or identified as outliers.

## RESULTS

**Figure 1:**
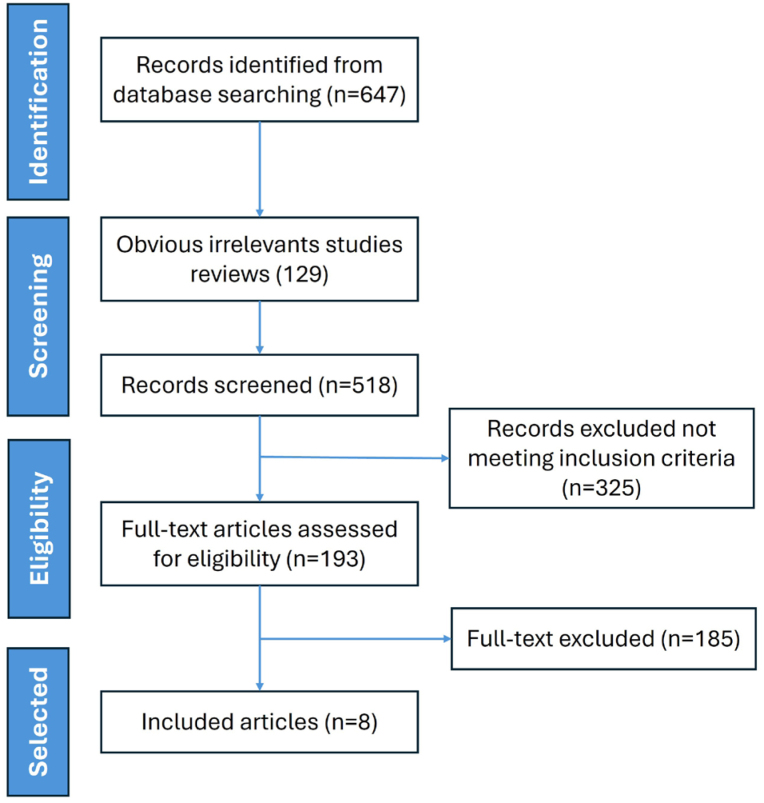
Flowchart of the selection process for the 8 studies included.

A comprehensive overview of the search strategy is presented in the PRISMA flowchart (Figure 1), highlighting the stepwise process of study retrieval and inclusion for this systematic review. A total of 647 articles were identified through electronic database searches using predefined search terms. Upon removal of duplicates, review articles, editorials, commentaries, case reports, or abstracts lacking sufficient data, 193 articles underwent abstract screening and full-text review, with selection based on the investigation of the association between BIR and AD. Ultimately, eight studies met the inclusion criteria and were selected for data extraction and analysis.

All selected studies explored the link between INI and AD. Of the eight studies that met the inclusion criteria, all were case-control studies. The studies were conducted across various countries, with sample sizes ranging from 35 to 538 participants.

### Relevant characteristics of the included studies

Table 1 ^13-20^ provides an overview of the included studies, and Table 2 ^13-20^ provides general characteristics of the studies included.

**Table 1:** Overview of the included studies.

Study	Type of study	Objective	Participants	Conclusion
Craft et al.^13^	RCT	"To examine the feasibility, safety, and efficacy of INI for the treatment of MCI and AD dementia in a phase 2/3 multisite clinical trial."	289 cases/249 placebo	"No cognitive or functional benefits were observed with INI treatment over a 12-month period among the primary intention-to-treat cohort."
Kellar et al.^14^	RCT	"To assess INI effects on cerebral spinal fluid markers of inflammation, immune, and vascular functions and their associations with clinical markers of AD progression."	24 cases/25 placebo	"INI altered the typical progression of markers of inflammation and immune response, suggesting compensatory immune response associated with therapeutic benefit."
Rosenbloom et al.^15^	RCT	""To evaluate safety and efficacy of rapid-acting intranasal glulisine in amnestic MCI or mild probable AD."	19 cases/16 placebo	"No enhancing effects of intranasal glulisine on cognition, function, or mood, but the ability to detect significance was limited by the number of subjects successfully enrolled and the study duration."
Kellar et al.^16^	RCT	"To assess INI effects on white matter health and its association with cognition and cerebral spinal fluid biomarker profiles in adults with MCI or AD in secondary analyses from prior phase 2 clinical trial data (NCT01767909)."	20 cases/16 placebo	"INI treatment for 12 months reduced white matter hyperintensity volume progression, supporting potential as a therapeutic option for AD."
Craft et al.^17^	RCT	"To examine INI effects on cognition, function, cerebral glucose metabolism, and cerebrospinal fluid biomarkers in adults with amnestic MCI or AD."	104 cases/66 placebo	"The results support longer trials of INI therapy for patients with amnestic MCI and patients with AD."
Craft et al.^18^	RCT	"To assess whether four months of INI detemir or regular insulin improves cognition, daily functioning, and AD biomarkers for adults with MCI or AD."	24 cases/12 placebo	"Future research warranted to explore mechanisms of treatment differences, and to further assess INI efficacy and safety."
Claxton et al.^19^	RCT	"To evaluate safety and efficacy of two insulin detemir doses for adults with AD or amnestic MCI compared with placebo."	40 cases/20 placebo	"Daily treatment with 40 IU insulin detemir modulated cognition for adults with AD or amnestic MCI, with APOE-related treatment response differences for the primary memory composite."
Claxton et al.^20^	RCT study	"To evaluate gender and ApoE genotype differences in response to two doses of INI in adults with MCI or AD."	74 cases/30 placebo	"Evidence suggests INI is a safe and effective for memory loss associated with MCI and AD, with variations by gender and ApoE ε4 carriage."

Abbreviations: RCT, randomized clinical trials; MCI, mild cognitive impairment; AD, Alzheimer disease; INI, intranasal insulin.

**Table 2:** General characteristics of the studies included.

Author	Year	Study country	Study type	Total case	Total placebo	% Gender M/F	Mean age (year) case/control	Insulin type	Daily dose UI	Study duration (days)
Craft et al.^13^	2020	USA	RCT	289	249	62.0/38.0	72.95/70.58	Humulin-R-100 Lilly	80	504
Kellar et al.^14^	2022	USA	RCT	24	25	55.0/45.0	69.94/71.58	Regular Insulin	20 or 40	434
Rosenbloom et al.^15^	2021	USA	RCT	19	16	57.1/42.9	63.2/79.0	Glulisine	20	224
Kellar et al.^16^	2021	USA	RCT	20	20	47.0/53.0	71.69/70.88	Humulin-R U-100	20	365
Craft et al.^17^	2012	USA	RCT	104	66	61,1/38.9	71.35/74.9	Regular insulin	20 or 40	120
Craft et al.^18^	2017	Canada	RCT	24	12	44.4/55.6	70.5/68.4	Insulin Determir/Regular Insulin	40	90
Claxton et al.^19^	2015	USA	RCT	40	20	-	-	Insulin Determir	20 or 40	21
Claxton et al.^20^	2013	USA	RCT	74	30	56.7/43.3	71.1/74.6	Novolin R	20 0r 40	120

Abbreviations: M, Male; F, Female; USA, United States of America; RCT, randomized clinical trials.

### Quality assessment

Systematic bias in research refers to any consistent error during data collection, analysis, interpretation, publication, or review process that leads to conclusions deviating from the truth. While RCTs are considered the gold standard in clinical research, they are prone to bias due to various factors such as sample selection and measurement variability.

**Figure 2:**
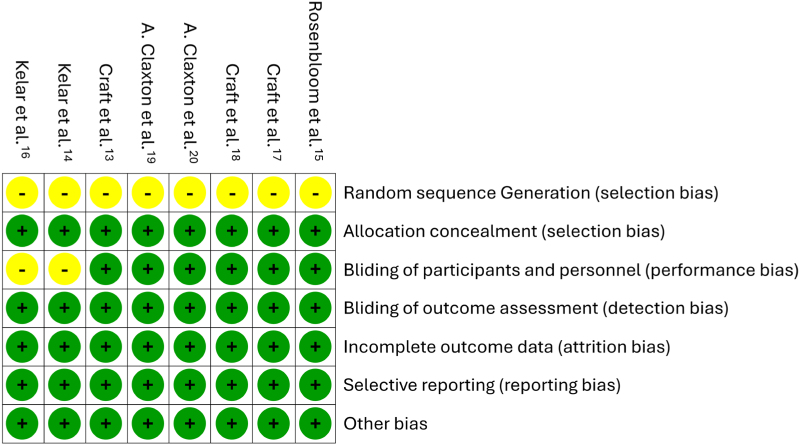
Risk of bias summary.

**Figure 3:**
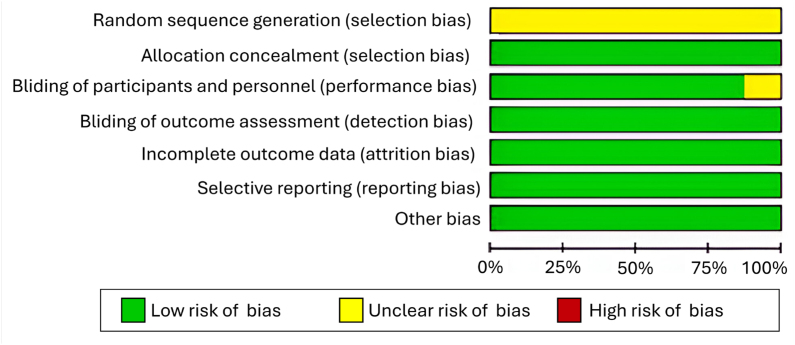
Risk of bias presented as percentage of the included studies.

A risk of bias assessment was conducted using the Cochrane Collaboration Network's RoB tool. Results indicated an overall low risk of bias, although uncertainties were identified in certain areas, including random sequence generation and participant blinding in some studies. Figure 2 ^13-20^ and Figure 3 ^13-20^ provided detailed assessments, with the crossover study by Rosenbloom et al.^15^ showing low risk, primarily due to its robust design.

### Meta-analysis of intranasal insulin for Alzheimer disease

This investigation employed a rigorous meta-analysis approach to assess the potential benefits of INI for AD. A comprehensive search strategy was implemented, encompassing all pertinent association studies published up to June 2024. To ensure inclusivity, a meticulous search was conducted across major electronic databases, including PubMed, Web of Science, Scopus, and Embase.

The meta-analysis identified eight original case-control studies examining the therapeutic response to INI in AD. Collectively, these studies encompassed a substantial sample size of 594 individuals.

**Figure 4:**
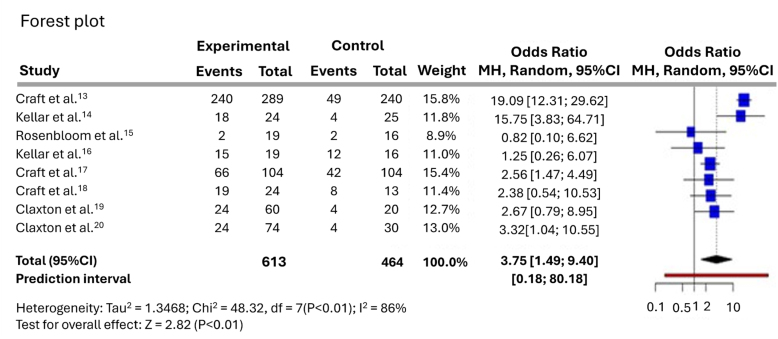
Comparative assessment of intranasal insulin *versus* placebo on global cognitive function in the overall study population.

A total of eight studies were analyzed, including 594 participants in the experimental cohort and 438 participants in the placebo cohort. Based on the analysis performed using random-effects model with Mantel-Haenszel method to compare the OR, a statistical difference was observed between the two cohorts. The overall OR was 3.75 with a 95%CI of 1.49–9.4. The test for overall effect showed significance at p<0.05. Significant heterogeneity was detected (0), suggesting inconsistency in the magnitude and/or direction of effects. The I^2^ value indicates that 85.5% of the variability among studies arises from heterogeneity rather than random chance (Figure 4).

**Figure 5:**
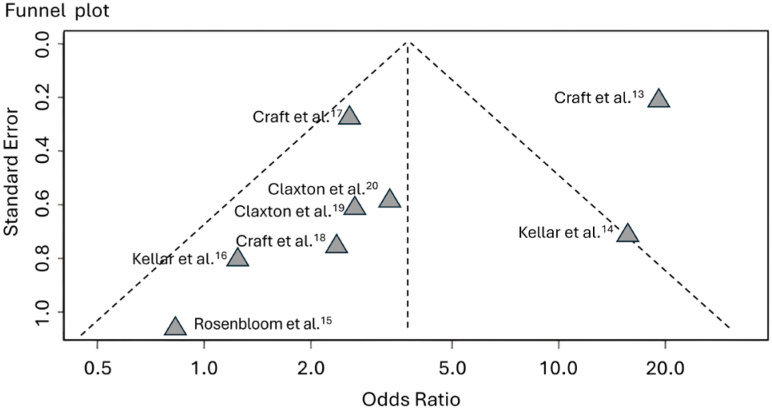
Funnel plot for meta-analysis.

The funnel plot does not indicate potential publication bias. The Eggers’ test does not support the presence of funnel plot asymmetry (intercept: −2.77, 95%CI −5.98 to 0.44, t: −1.689, p 0.142) (Figure 5).

The ADAS-Cog was employed to evaluate memory, language, functional ability, and attention. A smaller difference between scores indicated a stronger positive impact of the intervention on cognitive function. Comparative analysis of ADAS-Cog scores revealed a statistically significant superiority of INI over placebo, with standardized mean differences (SMD) of −1.59 (95%CI −2.91, −0.36) and SDM −1.50 (95%CI −2.61 to −0.28), respectively. Regarding the ADCS-ADL, the cognitive efficacy of INI at a dosage of 20 IU surpassed that of INI at 40 IU.

The discrepancy between the reported lack of cognitive improvement in the study by Craft et al.^13^ and its apparent contribution to a large OR for global cognitive improvement in the forest plot can be attributed to the specific methodology employed in the meta-analysis. While the study itself may not have observed a statistically significant difference in cognitive function, the meta-analysis incorporated a categorical measure of MCI that captured the overall trend or direction of change. Even when a single study does not show a significant effect, it can still contribute to the overall meta-analysis by providing additional data points. In this case, the OR associated with the study of Craft et al.^13^ likely reflects a trend toward improvement, even if statistical significance was no achieved.

## DISCUSSION

The results of this systematic review and meta-analysis provide evidence that INI administration significantly improves cognitive function in individuals with AD compared to placebo. Additionally, INI demonstrates a favorable safety profile, with a low incidence of side effects.

Traditionally, the pancreas has been regarded as the sole source of insulin crossing the blood-brain barrier. However, recent evidence suggests that nasal epithelium and serous glands may also synthesize insulin. Nasally derived insulin directly enters the central nervous system via the cribriform plate and travels along olfactory nerves to brain regions critical for learning and memory^21^. This insulin activates insulin-dependent functions involved in growth, metabolism, plasticity, survival, and cholinergic function within the brain^22^. A growing body of research has linked impairments in insulin receptor signaling in the development of dementia, including AD^23^.

The identification of insulin expression and secretion capabilities within the nasal mucosa suggests a physiological route for insulin delivery to the brain. Consequently, elevating brain insulin levels has been demonstrated to enhance verbal declarative and hippocampal memory. In AD, insulin administration improves cognitive function and decelerates cognitive decline. These observations provide compelling support for the concept of INS-based therapy for AD, encompassing both preventive and treatment strategies^24^.

The ADAS-Cog stands as a reliable tool for evaluating AD progression. It encompasses items assessing language, memory, praxis, and orientation, with higher scores indicating a greater impairment. These items prove valuable not only in differentiating AD patients from healthy individuals but also in determining disease severity, particularly through the orientation section^25^. Furthermore, distinguishing between MCI and dementia is crucial. MCI represents a transitional stage between dementia and the age-related cognitive decline, with both amnestic and non-amnestic subtypes^26^. In this meta-analysis, two studies failed to identify statistically significant cognitive improvements following INI administration. However, the power to detect significant effects was limited by the small sample size and short duration of the included studies. This is consistent with findings from an earlier investigation, which demonstrated that APOEε4-positive patients and female participants exhibited poorer recall following INI administration compared to non-APOEε4 carriers and male participants^20^.

The ADCS-ADL is a widely employed instrument for evaluating functional status in individuals with AD. It assesses the ability to perform a comprehensive range of daily activities, with higher scores on the ADCS-ADL indicating better preservation of functional capacity and lesser degree of impairment in daily living skills^27^. In this investigation, no statistically significant disparity in ADCS-ADL scores were observed between insulin treatment groups and the placebo control group. However, Claxton et al.^20^. identified a sex-based discrepancy in ADCS-ADL scores, favoring females^27^. Additionally, Craft et al. reported significant differences in ADCS-ADL scores between insulin and placebo groups specifically for AD patients, but not for those diagnosed with amnestic MCI^17^.

The core pathophysiological processes underlying AD are primarily driven by the aggregation of three key proteins: beta-amyloid peptides, tau protein, and hyperphosphorylated tau^28^. Previously, insulin has been hypothesized to exert a protective effect by mitigating amyloid-beta peptides accumulation and reducing tau phosphorylation^29^. However, this potential benefit is hampered by the limited ability of insulin to penetrate the blood-brain barrier^30^. To circumvent this obstacle, INI administration has emerged as a novel therapeutic strategy, leveraging the olfactory and trigeminal nerve pathways to bypass the blood-brain barrier via perivascular transport mechanisms^31^. Studies included in this meta-analysis evaluated the impact of INI on the cerebrospinal fluid concentrations of three important AD biomarkers: Aβ peptides, tau protein, and hyperphosphorylated tau. Interestingly, two studies by the same author reported discordant findings regarding the effects of INI administration^17,18^.

An essential advantage of intranasal administration lies in its ability to facilitate the direct delivery of large and charged therapeutic agents to the central nervous system via the nasal mucosa. This method bypasses the blood-brain barrier, thereby minimizing systemic exposure and mitigating the potential for adverse side effects associated with various brain-targeted therapies, including those that can otherwise penetrate the blood-brain barrier^32^. The meta-analysis evaluated the safety profile of INI administration and revealed no statistically significant difference in the incidence of adverse events between the insulin and placebo groups. All the included studies did not report any serious adverse effects. Minor complications were primarily limited to upper respiratory symptoms and rhinitis. While Rosenbloom et al.^15^ reported a higher incidence of nasal irritation, and Craft et al.^13^ noted a slightly higher total number of minor adverse events, the overall findings consistently demonstrated no significant difference in complication rates between the insulin and placebo groups. Therefore, when carefully considering the risk-benefit profile of this treatment modality, INI emerges as a potentially safe therapeutic option for patients with AD.

Various antidiabetic agents, including INI, have been explored for AD management^33^. In this meta-analysis, INI doses of 20 IU and 40 IU were assessed, with the lower dose demonstrating greater efficacy in improving ADAS-Cog scores. However, our systematic review and meta-analysis were limited by small sample sizes, heterogeneity, and variability in study design and insulin types and dosages. It is worth noting that the study with the largest sample size showed lower benefits associated with the intervention when compared to the other studies included in this meta-analysis, suggesting that it may be an outlier. In this study, Craft et al.^13^. identified several limitations associated with the INI administration that may have contributed to the attenuated treatment effects observed in their study. These limitations included challenges related to patient adherence, variations in dosing, and technical difficulties with the nasal delivery system. Furthermore, the study design may not have adequately accounted for potential variations in nasal anatomy or mucociliary clearance, which could have impacted the absorption of INI and its subsequent bioavailability. Despite these limitations, the findings provide evidence supporting the safety and efficacy of INI in this patient population.

In conclusion, this systematic review and meta-analysis evaluated the effects of INI administration in individuals with AD. While the data are not yet sufficient to establish INI as a definitive treatment for AD, the accumulating evidence supporting its safety, efficacy, and reduced systemic side effects strongly suggests that INI is associated with an overall enhancement of global cognition. The study's findings demonstrated a significant improvement in ADAS-Cog scores, particularly in participants receiving a 20 IU INI dosage. Furthermore, the observed adverse effects were minimal. Given the nascent nature of this research field, further studies are warranted to elucidate the heterogeneity in treatment responses to INI and to explore its potential cognitive benefits for a broader range of AD patients, aiming to improve their quality of life.
